# Are online symptoms checkers useful for patients with inflammatory arthritis?

**DOI:** 10.1186/s12891-016-1189-2

**Published:** 2016-08-24

**Authors:** Lucy Powley, Graham McIlroy, Gwenda Simons, Karim Raza

**Affiliations:** 1 Department of Rheumatology, Sandwell and West Birmingham Hospitals NHS Trust, Dudley Road, Birmingham, West Midlands B18 7QH UK; 2 Institute of Inflammation and Ageing, University of Birmingham, Birmingham, B15 2TT UK

**Keywords:** Inflammatory arthritis, Arthralgia, Internet, Symptom checker

## Abstract

**Background:**

Online symptom checkers are increasingly used by patients however there is little published evidence of their effectiveness in real patients. The aim of this study was to evaluate how patients with inflammatory arthritis and inflammatory arthralgia use the internet to look for health information and to assess the advice given and diagnoses suggested by the NHS and WebMD symptom checkers in relation to the patients’ actual diagnoses.

**Methods:**

Thirty-four patients with inflammatory arthritis (rheumatoid arthritis (*n* = 13), psoriatic arthritis (*n* = 4), unclassified arthritis (*n* = 4)) and inflammatory arthralgia (*n* = 13) newly presenting to a secondary care based clinic were identified using a consecutive sampling approach. Consenting patients were asked questions about their internet use in relation to their presenting symptoms. They then completed the NHS and the WebMD symptom checkers and their answers and the outcomes were recorded.

**Results:**

Sixteen patients had previously consulted the internet regarding their symptoms. Neither age nor gender significantly influenced internet usage. Actions advised via the NHS symptom checker were: call an ambulance (*n* = 11), attend A&E (*n* = 4), contact your GP straight away (*n* = 2), see your GP today (*n* = 6), or see your GP within 36 h (*n* = 11). The 5 most common differential diagnoses given by Web MD were gout (*n* = 28), rheumatoid arthritis (*n* = 24), psoriatic arthritis (*n* = 22), osteoarthritis (*n* = 18) and finger dislocation (*n* = 10). The most common first differential diagnosis was osteoarthritis (*n* = 12). Only 4 out of 21 patients with inflammatory arthritis were given a first diagnosis of rheumatoid arthritis or psoriatic arthritis.

**Conclusions:**

Our data highlight that help seeking advice given online is often inappropriate and that the diagnoses suggested are frequently inaccurate. Recommendations to seek emergency advice may cause inappropriate healthcare utilization.

**Electronic supplementary material:**

The online version of this article (doi:10.1186/s12891-016-1189-2) contains supplementary material, which is available to authorized users.

## Background

The early introduction of disease modifying anti-rheumatic drugs (DMARDs) leads to significantly improved outcomes for patients with rheumatoid arthritis (RA); in particular the first 12 weeks after symptom onset represent an important therapeutic window [1]. Despite this, delays still occur between symptom onset and the initiation of therapy [2, 3]. In particular, patients often wait for prolonged periods before seeking advice from healthcare professionals [2, 3]. Reasons for patient delay include a lack of knowledge about inflammatory arthritis and available therapies, a normalisation of symptoms and attribution to external factors [4–6].

Health information is increasingly obtained via the internet [7]. For example, Europe’s most popular health website, NHS choices [8], received over 10 million visits per week between January and October 2015 [9]. One way in which RA patients might try to determine whether they need to seek medical attention for the early symptoms of RA is by seeking information online. Indeed a recent UK survey of patients with newly presenting RA and unclassified arthritis showed that 37 % consulted the internet about their symptoms before seeking help from their GP (manuscript submitted).

Symptom checker type websites include triage tools which direct the user toward the most appropriate source of health care, and self-diagnosis tools which provide the user with a specific diagnosis or series of differential diagnoses. The NHS symptom checker and the Boots WebMD symptom checkers are well known and widely used online resources. In December 2014 they were the two highest ranked tools in the Google, Yahoo and Bing search engines when the search terms ‘symptoms checker’ were entered. Furthermore in December 2014, the NHS symptom checker received 721,517 visits (personal communication from Health and Social Care Information Centre) and the Boots WebMD website received 422,300 visits [10].

These types of diagnostic online tools are marketed as a way of saving patients time, decreasing anxiety and allowing patients to take control of their own health [11]. Many of these tools are designed to confer credibility by the use of symbols associated with the medical profession (e.g. stethoscopes) or by titles which infer that they can be used as proxies for doctors [11].

A recent study using 45 standardised patient vignettes to evaluate the accuracy of 23 symptom checkers found that they provided the correct diagnosis first on 34 % of occasions and listed the correct diagnosis within the top 20 differential diagnoses 58 % of the time. WebMD specifically provided the correct diagnosis 36 % of the time and listed it the top 20 62 % of the time [12]. However, in that study, researchers inputted data into the symptom checkers based on information within the vignettes; whether patients would have interpreted their symptoms and answered the questions in the same manner as researchers did is unclear. WebMD was evaluated using patients in an ENT clinic where it provided the correct diagnosis first in only 16 % of cases but included the correct diagnosis in the list in 70 % of cases [13].

Research evaluating web-based tools typically use a range of methods. For example a study looking at the usability of ANSWER, a patient decision aid, used rating scales and thought verbalisations completed by the patients with RA and observational data provided by the researchers [14]. Another study using ‘think-aloud’ protocols looked at how older adults search for information online and interpret symptoms using Google and WebMD’s symptom checker [15]. In that study, participants received a vignette depicting symptoms of illness and were asked to talk out loud about their thoughts and behaviour while they were trying to diagnose the problem using internet tools.

The diagnostic accuracy of symptom checkers in relation to the symptoms of inflammatory arthritis and arthralgia has not been widely evaluated. The current study used patients with inflammatory arthritis or inflammatory arthralgia rather than symptom vignettes to test both the NHS Choices symptom checker and the Boots WebMD symptom checker and evaluated the advice given in relation to the patient’s actual clinical diagnosis.

## Methods

### Participants

Newly presenting patients with either clinically apparent synovitis or a new onset of symptoms consistent with inflammatory arthritis but without clinically apparent synovial swelling (from here on referred to as inflammatory arthralgia) were recruited from a secondary care based rheumatology clinic between the 9/2/15 and the 27/7/15. All eligible patients who attended clinic between these dates were approached to participate.

### Assessment of internet use in relation to symptoms

Patients were asked if they had previously used the internet to find information about their presenting symptoms, which sites they had accessed and whether they had used symptom checkers.

### Assessment of the NHS and the Boots WebMD checker

Both the NHS and the Boots WebMD checker begin by requesting demographic data. The NHS symptom checker then asks patients to select a category that best describes their symptoms and goes on to ask a series of multiple choice questions. The questions asked depend on the answers to previous questions therefore not all patients are asked the same questions. At the end, patients are advised where they should seek healthcare. WebMD features a pictorial representation of the human body, and patients are asked to identify the areas where their symptoms are located. They are then invited to identify relevant symptoms (e.g. joint pain or swelling) and, depending on the symptoms selected, a series of multiple choice questions are asked. At the end, a list of differential diagnoses is produced.

Patients were asked to complete the NHS and Boots WebMD symptom checkers and their answers and the outcomes given by the symptom checkers (help-seeking advice given by the NHS symptom checker and the 5 most likely diagnoses identified by Web MD) were recorded. The advice given or diagnoses suggested by the symptom checkers were compared with classified diagnoses made at the patient’s assessment in secondary care.

### Clinical data

Data routinely collected during the new patient clinic included demographic variables, swollen and tender joint counts, DAS28 score, ESR, CRP, rheumatoid factor (RF) anti-CCP antibody status and rheumatological diagnosis according to established classification criteria. Autoantibody levels were considered to be ‘negative’ when they were below the upper limit of the normal reference range (ULN), ‘low’ when above the ULN but less than 3 times the ULN, and ‘high’ when over 3 times the ULN.

### Statistical analysis

Median and interquartile ranges were calculated for the continuous variables, and groups were compared using non-parametric tests. Differences between proportions were assessed with chi-squared tests. Significance was determined when *p* < 0.05. Data were analysed using SPSS version 20.

## Results

### Participants

Of the 35 patients approached to participate in this survey, 34 agreed. Participants had either a diagnosis of rheumatoid arthritis (*n* = 13), psoriatic arthritis (*n* = 4), unclassified inflammatory arthritis (*n* = 4) or inflammatory arthralgia (*n* = 13); their clinical characteristics are summarised in Table 1. The four groups were broadly comparable although there were differences in swollen joint counts and DAS28 scores – explained by the fact that patients in the inflammatory arthralgia group had no swollen joints.

**Table 1:** Characteristics of participants. Summary values are shown as median (interquartile range)

	Rheumatoid arthritis	Psoriatic arthritis	Unclassified arthritis	Inflammatory arthralgia	*p* for difference between groups
Number	13	4	4	13	
Sex	10 female	3 female	1 female	9 female	*p* = 0.27
Age (years)	52 (39–61.5)	47 (29.75–57.5)	69 (51.25–78.5)	42 (35–57.5)	*p* = 0.13
Tender joint count (out of 28)	8 (4.5–12)	5.5 (3.25–8.5)	3.5 (2.25–5.5)	3 (0–12)	*p* = 0.12
Swollen joint count (out of 28)	5 (1.5–5.5)	3 (1.25–6.25)	3 (1.5–5.25)	0	*p* < 0.001
Patient global score (out of 100)	49 (29–68.5)	48.5 (29.75–82.25)	19.5 (15.25–43.25)	46 (13.5–60.5)	*p* = 0.38
ESR (mm/h)	18 (11–48.5)	25.5 (12–33.75)	22.5 (14.5–72.5)	15 (8–20.5)	*p* = 0.28
CRP (mg/l)	9 (4.5–13.5)	6.5 (1.5–28)	4.5 (1.5–9)	2 (1–7)	*p* = 0.22
DAS28	4.61 (4.17–5.73)	4.64 (4.13–5.26)	4.1 (3.8–4.7)	3.61 (2.18–4.70)	*p* = 0.016
Rheumatoid factor positivity	Negative 6	Negative 4	Negative 4	Negative 11	*p* = 0.13
	Low 3			Low 0	
	High 4			High 2	
Anti-CCP antibody positivity *	Negative 8	Negative 3	Negative 4	Negative 8	*p* = 0.092
	High 4	Low 1		High 5	

*Anti-CCP antibody test was not performed in 1 RA patient

### Previous use of the internet to gather information about symptoms

Sixteen patients (47 %) had previously consulted the internet regarding their symptoms. There was no significant effect of age (median age 44.5 vs 55.5 years for patients who had vs patients who had not consulted the internet *p* = 0.164, Mann–Whitney *U* test) or gender (36 % of men and 52 % of women used the internet, *p* = 0.388, Chi square test) on internet usage. Two patients recollected having previously used NHS choices, and one recollected having previously used WebMD.

### NHS choices

**Fig. 1:**
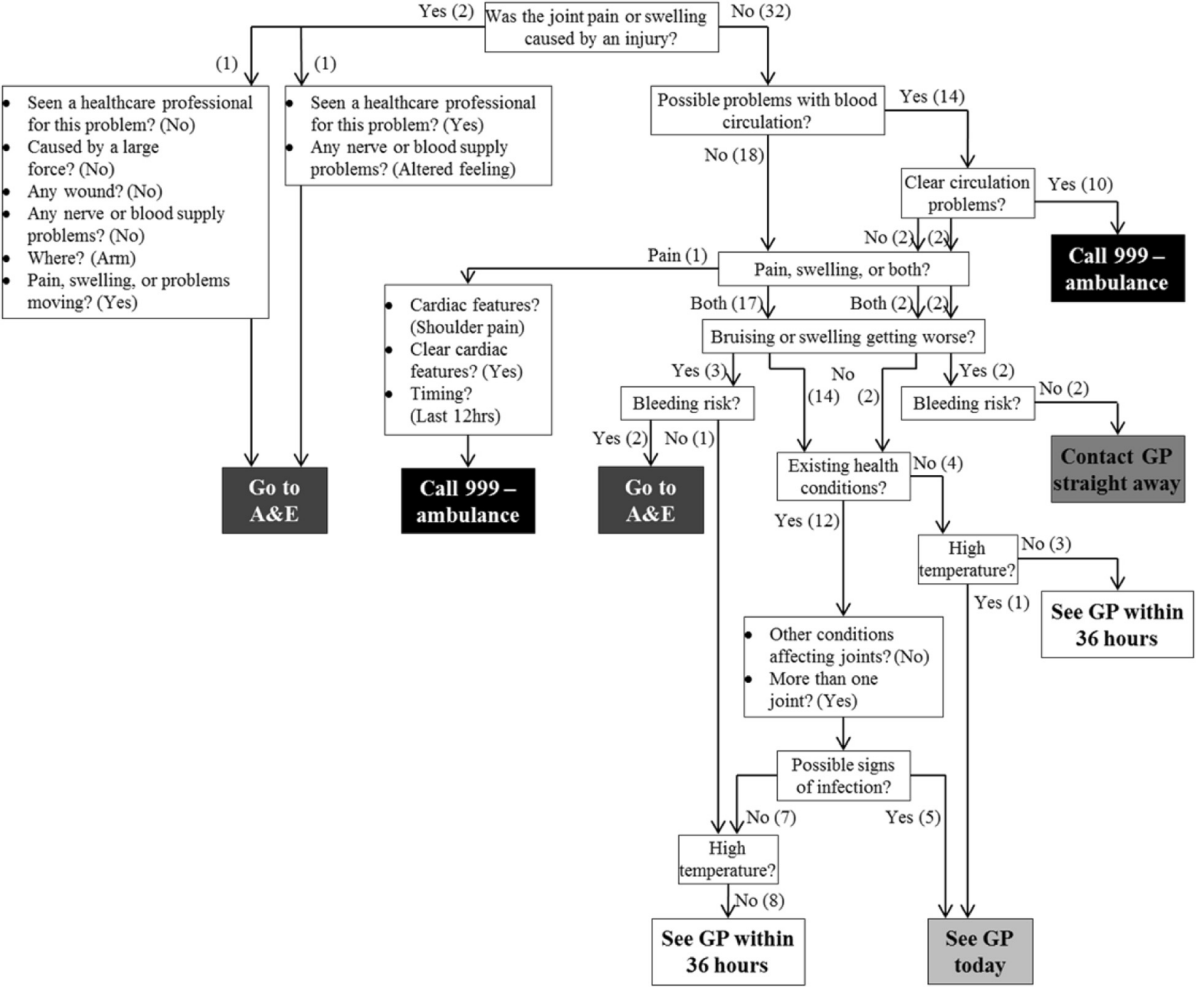
Responses given by patients when completing the NHS choices questionnaire and the recommendations given

The responses given by patients completing the NHS Choices symptom checker are summarised in Fig. 1. When asked to choose an initial category all patients chose joint pain and swelling. Actions advised were: call an ambulance (*n* = 11), attend A&E (*n* = 4), contact your GP straight away (*n* = 2), see your GP today (*n* = 6) or see your GP within 36 h (*n* = 11). There was no significant difference between the four diagnostic groups in the advice given by the NHS Choices symptom checker (*p* = 0.61, Chi square test). Advice to call an ambulance was driven by individuals responding to questions in a way which indicated that their symptoms were due to an acute cardiac (*n* = 1) or other circulatory (*n* = 10) problem.

### WebMD

**Fig. 2:**
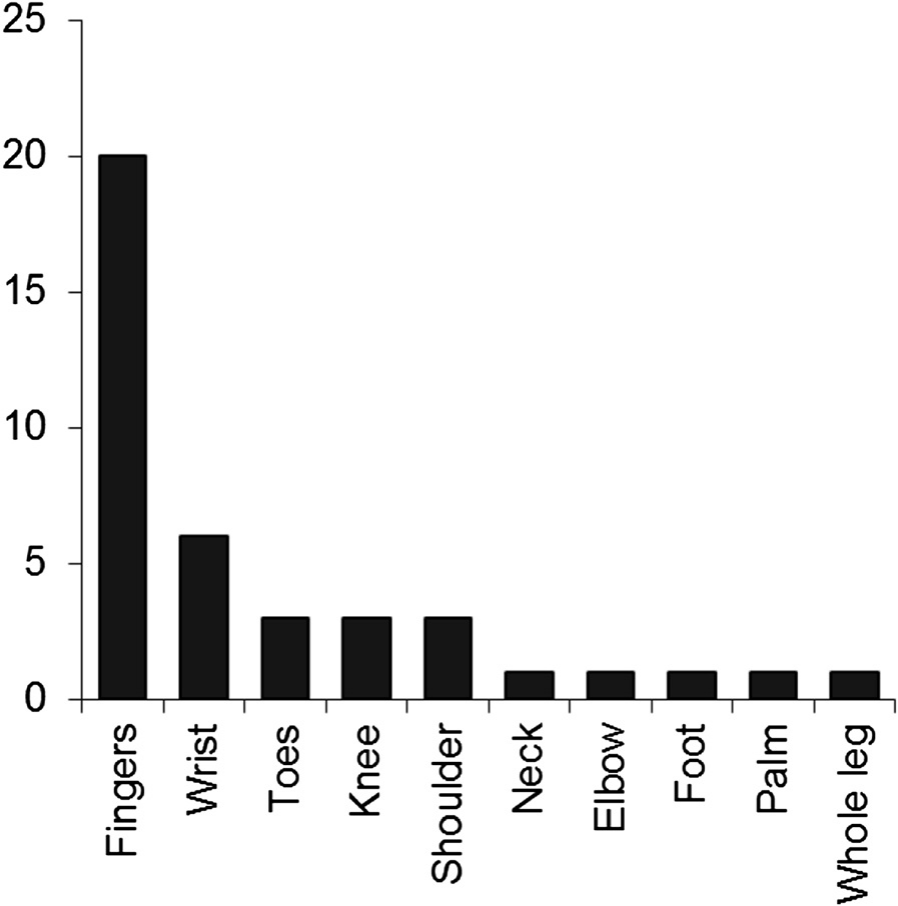
Frequency of affected body parts chosen by patients when using WebMD

**Fig. 3:**
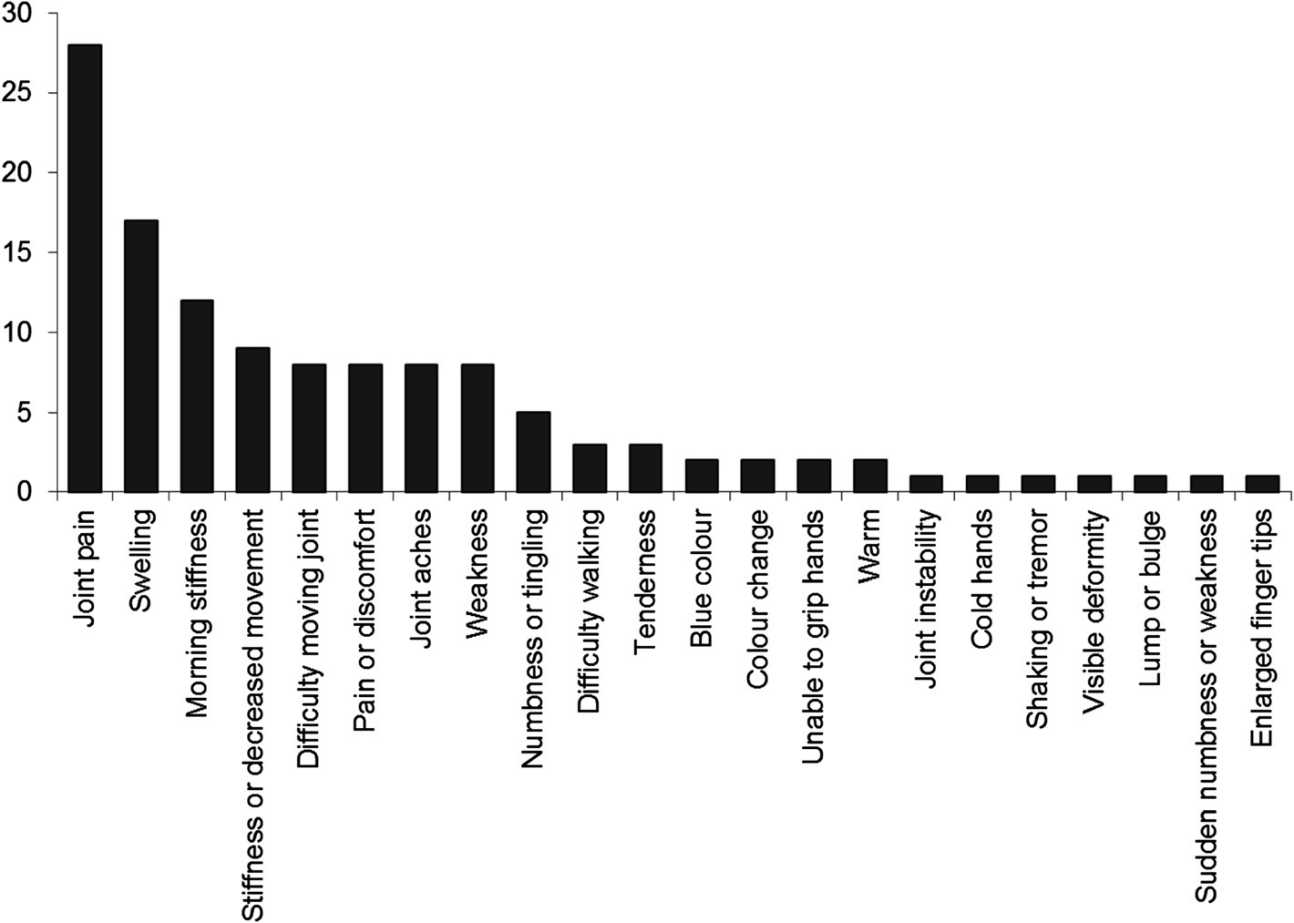
Frequency of symptoms chosen by patients when completing the WebMD symptom checker

**Fig. 4:**
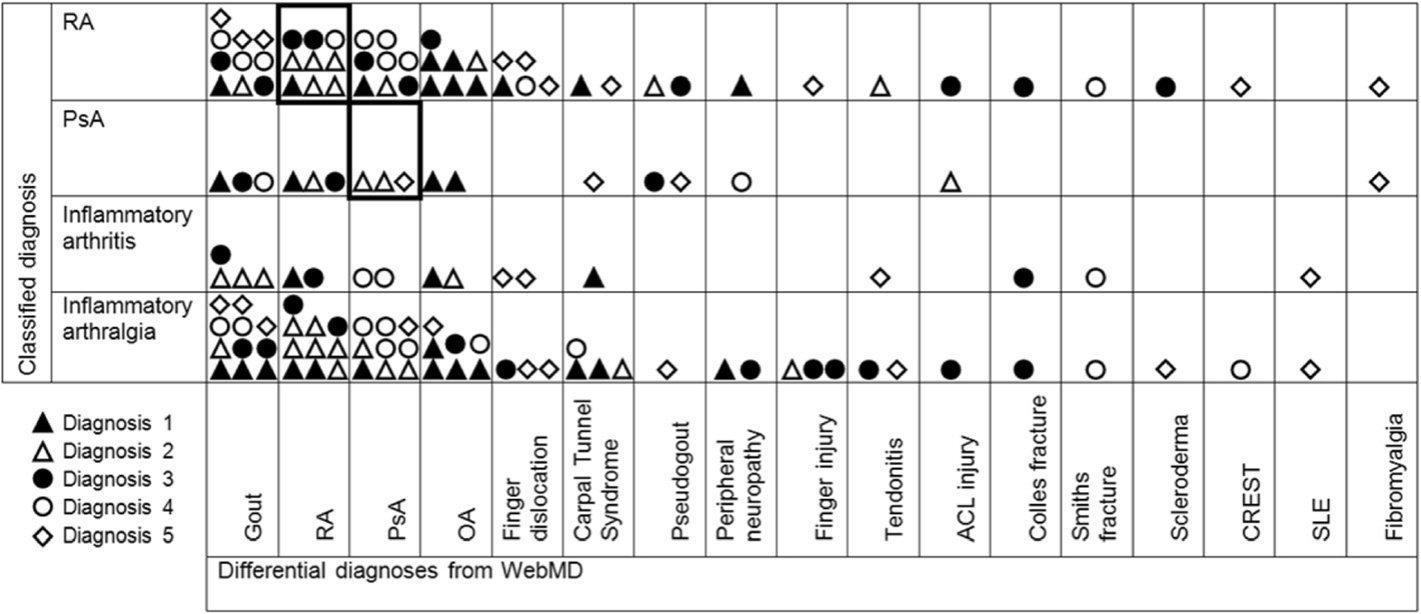
Differential diagnoses given by WebMD compared with diagnoses made in clinic according to established classification criteria. Different symbols reflect the 1st, 2nd, 3rd, 4th and 5th diagnoses given by WebMD as shown in the key within the Figure. Only diagnoses given by Web MD to more than 1 patient are shown. Twenty-seven additional diagnoses were listed once by WebMD, these are shown in the Additional file 1. *RA* rheumatoid arthritis, *PsA* psoriatic arthritis, *OA* osteoarthritis, *ACL* anterior cruciate ligament, *CREST* CREST syndrome (Calcinosis, Raynaud’s phenomenon, Esophageal dysmotility, Sclerodactyly, and Telangiectasia), *SLE* systemic lupus erythematosus

Whilst most patients only identified one body area, seven chose more than one. The frequency of the different body areas selected is shown in Fig. 2 with the most commonly selected area being the fingers (59 % of patients). Joint pain (82 %), swelling (50 %) and morning stiffness (35 %) were the most commonly reported symptoms (Fig. 3). Gout appeared most commonly in the top 5 differential diagnoses (*n* = 28), followed by RA (*n* = 24), psoriatic arthritis (PsA) (*n* = 22), osteoarthritis (*n* = 18) and finger dislocation (*n* = 9). Osteoarthritis was the most common top differential diagnosis, and RA appeared most often as the second differential diagnosis. Only 4 out of 21 patients with inflammatory arthritis were given a first diagnosis of RA or PsA by WebMD. Only 69 % of RA patients and 75 % of PsA patients had their actual diagnosis listed amongst the top 5 differential diagnoses given by WebMD. A comparison between the most common differential diagnoses given by WebMD and clinical diagnoses is illustrated in Fig. 4. A list of differential diagnoses given by WebMD to less than 2 patients can be found in Additional file 1.

## Discussion

This study found that the help seeking advice and diagnoses given by the symptom checkers tested was frequently inaccurate. For example, the NHS symptom checker inappropriately suggested that nearly half of patients should seek advice from emergency services. Only 56 % of patients were advised to seek help from primary care which would have been the appropriate triage level for their condition. Whilst we recognise the need for symptom checkers to try and rule out conditions requiring urgent medical treatment, were patients with inflammatory arthritis to follow the advice from the symptom checker it would result in poor use of emergency healthcare resources.

Previous studies have also suggested that symptom checkers tend to be risk adverse either advising patients to seek medical care when self-care would be appropriate or advising patients to attend hospital rather than consult a primary care physician [12]. A study which used patient vignettes to test 15 different symptom checkers which gave triage advice found appropriate triage advice was only being given in 57 % of cases [12].

The diagnostic information provided by WebMD was mixed. Whilst only 4 out of 21 patients with inflammatory arthritis were given a first diagnosis of PsA or RA, these diagnoses were listed in the top 5 differentials in 15 out of 21 patients. However, osteoarthritis was suggested as the first differential diagnosis in 8 patients with inflammatory arthritis (5 with RA, 2 with PsA and 1 with an unclassified arthritis). Given that self-management approaches are frequently promoted as initial management strategies for patients with osteoarthritis this may delay appropriate help seeking for these patients with inflammatory arthritis.

### Strengths and limitations

This study only evaluated symptom checker use in patients with inflammatory joint disease therefore our conclusions may not be generalizable to patients with other conditions. Although the sample size is small, the methodology of the study was more robust than many studies which have evaluated symptoms checkers as it used real patients rather than vignettes.

## Conclusions

The use of online resource by patients in the context of their symptoms is commonplace and likely to become more frequent over time. This study highlights that, in the context of inflammatory arthritis symptom, triage advice from the NHS Choices and WedMD checkers is risk averse and often inappropriate. Whilst the accuracy of the first suggested diagnosis is poor the list of differentials does often contain the correct diagnosis.

## Abbreviations

ACL, anterior cruciate ligament; CCP, cyclic citrullinated peptide; CREST, Calcinosis, Raynaud’s phenomenon, Oesophageal dysmotility, Sclerodactyly, and Telangiectasia; CRP, C reactive protein; DAS 28, disease activity score 28 joints using erythrosedimentation rate; DMARD, disease modifying antirheumatic drug; ESR, erythrocyte sedimentation rate; OA, osteoarthritis; PsA, psoriatic arthritis; RA, rheumatoid arthritis; RF, rheumatoid factor; SLE, systemic lupus erythematosus; ULN, upper limit of normal

## Additional files

Additional file 1: List of differential diagnoses given by WebMD to only 1 patient. (DOCX 13 kb) Additional file 2: Dataset used for this study. (XLSX 70 kb)
