# Supplementary material for: Are online symptoms checkers useful for patients with inflammatory arthritis?
Source: BMC Musculoskelet Disord. 2016 Aug 24;17(1):362. doi: 10.1186/s12891-016-1189-2 (PMC4995741; doi:10.1186/s12891-016-1189-2)
Supplement: Additional file 1: — List of differential diagnoses given by WebMD to only 1 patient. (DOCX 13 kb) [file 12891_2016_1189_MOESM1_ESM.docx]

**Table S1 – List of differential diagnoses given by WebMD to only 1 patient**

| Muscle Strain |
| --- |
| Finger infection |
| Paronychia |
| Dislocated shoulder |
| Frozen shoulder |
| Rotator cuff injury |
| Collar bone fracture |
| Septic Arthritis |
| Shingles |
| Sciatica |
| Poorly fitting shoes |
| Foot fracture |
| Lumbar spine stenosis |
| Coronary artery disease |
| Osteomyelitis |
| Repetitive motion injury |
| Patello femoral pain syndrome |
| Saturday night palsy |
| Abscess |
| Toe fracture |
| Elbow fracture |
| Tennis elbow |
| Bee/wasp sting |
| Insect bite |
| Insect sting |
| Multiple sclerosis |
| Cervical Spine stenosis |
